# Modifying Serum Plant Sterol Concentrations: Effects on Markers for Whole Body Cholesterol Metabolism in Children Receiving Parenteral Nutrition and Intravenous Lipids

**DOI:** 10.3390/nu11010120

**Published:** 2019-01-08

**Authors:** Jogchum Plat, Sabine Baumgartner, Anita C.E. Vreugdenhil, Maurice C. J. M. Konings, Kara L. Calkins, Ronald P. Mensink

**Affiliations:** 1 Department of Nutrition and Movement Sciences, School of Nutrition and Translational Research in Metabolism (NUTRIM), Maastricht University, 6229ER Maastricht, The Netherlands; sabine.baumgartner@maastrichtuniversity.nl (S.B.); maurice.konings@maastrichtuniversity.nl (M.C.J.M.K.); r.mensink@maastrichtuniversity.nl (R.P.M.); 2 Department of Pediatrics, School of Nutrition and Translational Research in Metabolism (NUTRIM), Maastricht University, 6229ER Maastricht, The Netherlands; A.vreugdenhil@mumc.nl; 3 Department of Pediatrics, Division of Neonatology and Developmental Biology, Neonatal Research Center, David Geffen School of Medicine, University of California, Los Angeles, Mattel Children’s Hospital at UCLA, Los Angeles, CA 90095, USA; KCalkins@mednet.ucla.edu

**Keywords:** cholesterol synthesis, lathosterol, cholesterol absorption, cholestanol, plant sterols, intestinal failure associated liver disease, parenteral nutrition

## Abstract

Background: Non-cholesterol sterols are validated markers for fractional intestinal cholesterol absorption (cholestanol) and endogenous cholesterol synthesis (lathosterol). This study’s objective was to evaluate markers for cholesterol synthesis and absorption in children exposed to two different intravenous lipid emulsions that rapidly change serum plant sterol concentrations as part of their parenteral nutrition (PN). Methods: Serum samples from two different studies were used: (1) nine PN-dependent children with intestinal failure associated liver disease (IFALD) whose soy-based, plant sterol-rich lipid (SO) was replaced with a fish-based, plant sterol-poor (FO) lipid; and (2) five neonates prescribed SO after birth. In the first study, samples were collected at baseline (prior to FO initiation) and after 3 and 6 months of FO. In study 2, samples were collected at 1 and 3 weeks of age. Results: In study 1, a 7-fold reduction in campesterol, a 12-fold reduction in sitosterol, and a 15-fold reduction in stigmasterol was observed 6 months after switching to FO. Serum cholesterol concentrations did not change, but cholesterol-standardized lathosterol increased (3-fold) and cholesterol-standardized cholestanol decreased (2-fold). In study 2, after 3 weeks of SO, sitosterol and campesterol concentrations increased 4-5 fold. At the same time, cholesterol-standardized lathosterol increased 69% and cholesterol-standardized cholestanol decreased by 29%. Conclusion: Based on these finding we conclude that changes in serum plant sterol concentrations might have direct effects on endogenous cholesterol synthesis, although this needs to be confirmed in future studies. Moreover, we speculate that this changed synthesis subsequently affects intestinal cholesterol absorption.

## 1. Introduction

Studies in healthy subjects have shown that serum non-cholesterol sterol concentrations are biomarkers for fractional intestinal cholesterol absorption (sitosterol, campesterol, cholestanol) and endogenous cholesterol synthesis (lathosterol, desmosterol) [1]. Moreover, some plant sterols, specifically sitosterol, campesterol and stigmasterol, directly affect the expression of genes involved in cholesterol metabolism, i.e., the SREBP-2 [2,3,4] and LXR pathways [4,5,6]. In humans, cholesterol homeostasis is maintained by various feedback and compensatory mechanisms. As a result, it is difficult to detangle the direct and indirect effects of dietary plant sterol intake. A common assumption is that consuming foods enriched in plant sterols lowers intestinal cholesterol absorption. This occurs because sterols interfere with mixed micelle formation in the intestinal lumen, in turn lowering the bioavailability and flux of cholesterol from the intestinal lumen into the circulation and increasing hepatic cholesterol synthesis [7]. The net effect of reduced absorption and increased cholesterol synthesis is a reduction in serum low-density lipoprotein (LDL) cholesterol concentrations [8]. Simultaneously, dietary plant sterol consumption raises serum sitosterol and campesterol concentrations by approximately 30% [9]. Hence, since multiple metabolic pathways change, it is difficult to determine whether circulating plant sterols directly affect endogenous cholesterol synthesis. However, this is a clinically relevant question, because cholesterol precursors like desmosterol and mevalonate have amongst others clear effects on cells in our immune system [10,11]. 

To investigate whether plant sterols directly influence gene expression in vivo in humans, an experiment that alters plant sterol concentrations without affecting cholesterol fluxes from the intestinal tract would be required. Patients who rely upon parenteral nutrition (PN) for fluids, electrolytes, and nutrients generally absorb minimal nutrition from the intestine. These patients also depend on intravenous (IV) lipid emulsions for non-protein calories and fatty acids. Most lipid emulsions are soy-based, and provide a large source of intravenous plant sterols. Hence, this unique patient population provides an opportunity to study the effect of circulating plant sterols on endogenous cholesterol synthesis and intestinal cholesterol absorption. Therefore, the objective of this study was to investigate changes in serum markers that reflect endogenous cholesterol synthesis (lathosterol) and intestinal cholesterol absorption (cholestanol) in two different groups of children who received PN supplemented with two different IV lipid emulsions. In the first group, we studied PN-dependent children with intestinal failure-associated liver disease (IFALD) whose soy-based, sterol-rich lipid emulsion (SO) was replaced with a fish-based, sterol-poor lipid emulsion as a treatment for IFALD. Pediatric IFALD is a liver disease that occurs in children exposed to long-term PN. IFALD is initially characterized by cholestasis (conjugated hyperbilirubinemia) and periportal inflammation (hepatitis), and can progress to fibrosis, cirrhosis, and end-stage liver disease. Prolonged SO exposure leads to increased serum sterol concentrations that disturb bile acid metabolism causing IFALD [12]. In the second group, we studied neonates in the intensive care unit who received SO as part of their PN regimen who are at risk for PN-associated cholestasis. 

The objective of this study was to explore in children receiving PN supplemented with two different IV lipid emulsions whether rapid changes in plant sterol concentrations affects endogenous cholesterol synthesis while a second explorative objective was to evaluate whether in this particular IFALD population on PN nutrition, intestinal cholesterol absorption would follow. 

## 2. Materials and Methods

### 2.1. Subjects

This is an observational, hypothesis-generating study. In study 1, samples were collected from children who developed IFALD secondary to prolonged PN-dependence as a result of a congenital or acquired gastrointesintal disorder resulting in intestinal failure. Most of the subjects had short bowel syndrome (SBS). As part of a compassionate use protocol, SO (Intralipid^®^, Fresenius Kabi, Uppsala, Sweden) was replaced with FO (Omegaven^®^, Fresenius Kabi, Bad Homburg, Germany) as a treatment for IFALD (Table 1). FO was dosed at 0.5 g/kg/day IV for the first 2 days, then 1 g/kg/day IV thereafter over 8–24 h for 6 months [12]. Blood was collected at three time points, while receiving SO (prior to FO initiation), and at approximately 3 and 6 months of FO treatment. The primary medical team was responsible for prescribing THE subject’s enteral nutrition and PN, with the exception of FO, which was prescribed by the research team. At baseline, the mean (±SD) energy intake was 7% (±13%) and 93% (±13%) from enteral sources and PN, respectively. At 6 months, 13% (±24%) and 87% (±24%) of energy was obtained from enteral sources and PN, respectively (Table 2). Subjects who received enteral nutrition received either breast milk, infant elemental formulas, or oral electrolyte replacements. Parents/legal guardians provided written informed consent. The study was registered at clinicaltrials.gov as NCT00969332. 

The second study consisted of premature neonates or neonates with congenital gastrointestinal disorders who were hospitalized in the neonatal intensive care unit and received SO as part of their standard PN regimen (Table 1). SO was generally dosed at 0.5–1 g/kg/day IV on the first day of life, and advanced 0.5–1 g/kg/day IV to a maximum dose of 3 g/kg/day IV. In this study, blood was collected at study enrollment (during the first few days of life, days 2–5) and then again in the third week of life (days 21–25). At baseline, on average 1% (±3%) of energy was derived from enteral sources and 99% (±3%) from PN. After 3 weeks, intake increased to 51% (±47%) and 49% (±46%) from enteral nutrition and PN, respectively (Table 2). All subjects received maternal breast milk. Verbal consent was obtained from parents/legal guardians. Both studies were conducted according the ethical guidelines of the 1975 Declaration of Helsinki and approved by the institutional review board at University of California, Los Angeles. 

### 2.2. Sample Processing

Blood was collected in EDTA tubes and then centrifuged at 1300× *g* for 10 min at 4 °C to obtain plasma. To obtain serum, samples were allowed to clot for 1 h at 20 °C, and then centrifuged at 1300× *g* for 15 min at 20 °C. Plasma and serum supernatants were transferred into 1.5 mL Eppendorf tubes and stored at −80 °C for future use. 

#### 2.2.1. Biochemical Measurements

Plant sterols (sitosterol, campesterol and stigmasterol), cholestanol, a marker for intestinal cholesterol absorption, and lathosterol, a marker for endogenous cholesterol synthesis were analyzed by gas chromatography with flame ionization detection (GC-FID), as previously described [13]. Levels were expressed in absolute concentrations (µmol/L), as well as standardized for cholesterol concentrations (µmol per mmol total cholesterol). For standardization, absolute concentrations were divided by cholesterol concentrations from the same sample that were quantified in the same GC-FID run. All samples from one subject were analyzed within the same run.

#### 2.2.2. Statistics

Non-cholesterol sterols are reported as means ± SD. Concentrations at baseline, and after 3 months and 6 months of FO treatment (study 1) and baseline and approximately 3 weeks of age (study 2) were evaluated by a Wilcoxon signed rank test. Results were considered to be statistically significant if *p* < 0.05 and statistical analyses were performed using SPSS 20.0 for Mac Os X or higher (SPSS Inc., Chicago, IL, USA). 

## 3. Results

In study 1, 9 children (7 males, 2 females) were included. The median age at the start of FO was 6.8 months (range 1–99 months) (Table 2). All of the children had biochemical evidence of IFALD. Serum total and conjugated bilirubin concentrations and aspartate and alanine aminotransferase concentrations were abnormally high, indicating cholestasis and hepatocyte damage (Table 3). Serum sitosterol (319 ± 177 µmol/L), campesterol (96.6 ± 53.7 µmol/L) and stigmasterol (55.3 ± 36.2 µmol/L) concentrations of children in this study were extremely high at baseline. As expected, when compared to baseline, after 3 and 6 months of FO treatment, serum sitosterol, campesterol and stigmasterol concentrations dramatically decreased. As shown in Table 4, after 6 months of FO, sitosterol concentrations decreased ~12-fold to 27.1 ± 42.4 µmol/L (*p* < 0.05 vs. baseline); campesterol concentrations decreased ~7-fold to 14.6 ± 17.2 µmol/L (*p* < 0.05 vs. baseline); stigmasterol decreased ~15-fold to 3.5 ± 5.3 µmol/L (*p* < 0.05 vs. baseline). Serum plant sterol concentrations after 6 months of FO were comparable, but still slightly higher than the concentrations observed in neonates at baseline in study 2. Interestingly, despite the pronounced changes in serum non-cholesterol concentrations, there was no significant change in serum total cholesterol concentrations after 3 and 6 months of FO when compared to baseline (Table 4). Consequently, cholesterol-standardized sitosterol, campesterol and stigmasterol concentrations decreased at both time points when compared to baseline (*p* < 0.001).

In study 1, regarding changes in cholesterol metabolism, cholesterol-standardized lathosterol concentrations (reflecting whole body endogenous cholesterol synthesis) increased 3-fold, whereas cholesterol-standardized cholestanol concentrations (reflecting intestinal cholesterol absorption) decreased 2-fold when compared to baseline (*p* < 0.005) (Table 4). 

In study 2, 5 neonates (1 male, 4 females) were included in the study. Most neonates were premature, with a median gestational age of 29 weeks (range 25–37 weeks) (Table 2). At enrollment, the median age was 2 days (range 1–4 days). Serum sitosterol, campesterol and stigmasterol concentrations at 3 weeks of age were significantly higher when compared to concentrations at baseline. As shown in Table 5, sitosterol concentrations increased from 20.5 ± 13.9 to 91.8 ± 51.0 µmol/L (*p* < 0.05); campesterol concentrations increased from 6.1 ± 4.6 to 30.5 ± 19.6 µmol/L (*p* < 0.05); and stigmasterol concentrations increased from 4.1 ± 2.9 to 15.1 ± 8.4 µmol/L (*p* < 0.05). Interestingly, serum cholesterol concentrations decreased slightly, although this difference was not statistically significant. Consequently, cholesterol-standardized sitosterol, campesterol and stigmasterol concentrations at 3 weeks increased approximately 5-fold when compared to baseline (*p* < 0.05). 

In study 2, there was a 69% increase in cholesterol-standardized lathosterol concentrations and a 29% reduction in cholesterol-standardized cholestanol concentrations (Table 5). While cholesterol-standardized lathosterol increased, it did not reach statistical significance. However, the reduction in cholesterol-standardized cholestanol was statistically significant (*p* = 0.043).

## 4. Discussion

Earlier in vitro and ex vivo studies suggested that plant sterols such as sitosterol, campesterol and stigmasterol, directly affect the expression of genes involved in cholesterol metabolism [2,3,4,5,6]. Since in vivo plant sterol consumption not only increases plasma plant sterol concentrations, but also lowers serum cholesterol concentrations, changes in gene expression or other physiological responses cannot exclusively be ascribed to either an increase in plant sterol or a decrease in cholesterol levels. In this study, we took advantage of two groups of children that would be anticipated to have minimal intestinal cholesterol absorption. In study 1, children with intestinal failure received the vast majority of their calories from PN because of intestinal malabsorption. In study 2, premature neonates and neonates with congenital gastrointestinal disorders receive PN as their sole form of nutrition (PN) after birth and enteral feeds were slowly introduced. We showed that under two different parenteral feeding conditions, serum plant sterol (sitosterol, campesterol and stigmasterol) concentrations changed quite dramatically and quickly, whereas serum cholesterol concentrations remain rather stable. Serum plant sterols decreased 7–15 fold in study 1 and increased 4–5 fold in study 2. These changes reflect the sterol composition of the different IV lipid emulsions. As anticipated, the observed changes in serum plant sterols are larger as compared to the approximate 30% increase in serum plant sterol concentrations normally seen after consuming plant-sterol enriched diets [9]. These differences were anticipated, considering the mode of delivery (parenteral vs. enteral) and lipid type (SO vs. FO), and justified the choice of these unique conditions as an approach to study the direct effects of plant sterols on cholesterol metabolism. 

Our results suggest that plant sterols may have direct effects on cholesterol metabolism. This conclusion is based on changes in serum lathosterol and cholestanol levels that reflect changes in endogenous cholesterol synthesis and intestinal fractional cholesterol absorption, respectively. For example, in children whose SO was replaced with FO (study 1), plant sterol concentrations were reduced and endogenous cholesterol synthesis, as reflected by serum cholesterol-standardized lathosterol concentrations, increased. These results support the earlier observation by Hallikainen and coworkers [14], who described the characteristics in one adult with SBS. In our study, we describe the results of a larger cohort of children with IFALD (*n* = 9). This observation might suggest that the presence of increased plant sterol concentrations, i.e., after consumption of plant sterol-rich foods, would actually dampen endogenous cholesterol synthesis. This is why this current study is important, since we normally observe an elevation in endogenous cholesterol synthesis after plant sterol consumption. It is now tempting to speculate that this increase in cholesterol synthesis is a compensatory response to a lower cholesterol flux from the intestine and cancels out the potential direct effect of increased plant sterol concentrations that would actually reduce cholesterol synthesis. An explanation for this potential direct effect of plant sterols on cholesterol synthesis could be that intracellular cholesterol sensors like SREBP-2 and LXR may not be able to differentiate between the structure of cholesterol and a plant sterol molecule. Indeed, already in 1997, Field and Mathur showed in vitro in differentiated Caco-2 cells that adding sitosterol inhibited the SREBP-2 pathway thereby reducing HMG-CoA reductase expression [2]. The question remains how to explain that consumption of plant stanols or treatment with ezetimibe also elevates cholesterol synthesis, while plant sterol concentrations are reduced. This can be explained by two potential mechanisms, i.e., a compensatory response towards lower intestinal cholesterol absorption, or direct increased synthesis due to the fact that plant sterol concentrations are lowered by plant stanols and ezetimibe. 

Although this appears to be a straightforward explanation for direct effects of plant sterols on cholesterol synthesis, our results in neonates who are exposed to SO (study 2) complicate matters. In this study, serum plant sterol concentrations increased with SO, but also cholesterol-standardized lathosterol concentrations increased. These findings are in line with data from Kurvinen et al. in 2011 and 2012 [15,16], who concluded that high-serum plant sterols were associated with increased endogenous cholesterol synthesis. The strength of our study is that in contrast to the earlier studies by Kurvinen, our subjects served as their own controls, allowing us to compare changes at different time points. However, the reported changes in cholesterol synthesis in conditions where plant sterol concentrations are increased are not consistent, since others have reported unchanged [17] or even decreased synthesis [18] in these conditions. These discrepancies may be caused by differences in patient populations, i.e., comparisons of SBS patients with PN vs. SBS patients without PN, or versus healthy controls. Patients with SBS have higher rates of cholesterol synthesis [19], most likely because intestinal cholesterol absorption is minimal to zero. Additional reasons for this observed inconsistency may include: (1) differences in medical conditions (some patients still have parts of their gastrointestinal tract while others did not), (2) differences in feeding regimens (full PN vs. partial PN), (3) differences in the age of the subjects (neonate, child or adult), or (4) variation in follow-up duration. 

A final aspect to consider when interpreting our cholesterol synthesis data is the liver status of the children. In study 1, while plant sterol concentrations are lowered when switching to FO, IFALD biochemically resolves. Therefore, it is theoretically possible that the observed increased cholesterol synthesis is a compensatory response as part of tissue repair. 

An important part of cholesterol balance is regulated via intestinal cholesterol absorption, which normally shows the opposite of endogenous cholesterol synthesis [20]. This is logical since maintaining cholesterol balance demands cholesterol supplies from either one process but not both. This is also the basis behind the concept of identifying so-called cholesterol absorbers or cholesterol synthesizers [21]. Apparently, some individuals primarily obtain their cholesterol from synthesis (and consequently have a low intestinal absorption), whereas others rely on a high absorption and have a low synthesis [22,23]. Indeed, in study 1, switching from SO to FO significantly reduced serum plant sterol concentrations, increased cholesterol synthesis, and decreased intestinal cholesterol absorption as reflected by reduced cholestanol concentrations. This was also observed in study 2, where cholesterol synthesis was increased and cholestanol was decreased at the same time. The question is how to interpret the changes in serum cholestanol as a marker for intestinal cholesterol absorption. In patients with intestinal failure and a cholestatic liver, there is probably not much cholesterol in the intestinal lumen available for absorption. For example, in study 1, children had a cholestatic liver at baseline, which indicates that very little cholesterol will enter the intestinal lumen via biliary secretion. Via the diet a small amount of cholesterol was available since 7% of energy was coming via enteral sources at baseline, which was either breast milk, infant elemental formula, or oral electrolyte replacement. After 6 months on FO, IFALD resolved and the liver could secrete cholesterol into bile. At this time 13% of energy was from enteral nutrition. In other words, the supply of cholesterol into the intestinal lumen was greater at 6 months, and the reduction in cholesterol-standardized cholestanol concentrations, which is a marker for fractional intestinal cholesterol absorption, can either be a real reduction in absorption or an effect due to the larger supplies. In study 2, it might also be a cholesterol supply effect since the percent of energy from enteral sources elevated from 1 to 51 en%, which also indicates a larger enteral cholesterol intake. 

We recognize our study’s limitations. Our sample size is small. Moreover, enteral intake changed over time, and subjects received different types of enteral nutrition. While in study 2, all of the subjects received breast milk, two subjects were weaned from PN at the end of the study. Moreover, we can only comment on associations considering our study’s design. Last, while we focused on PN-induced changes in plant sterol concentrations, there are several other possible explanations for the change in liver parameters in study 1. FO contains high concentrations of omega-3 polyunsaturated fatty acids and the antioxidant Vitamin E that also contribute to cholestasis resolution [12]. The question is whether the observed changes in endogenous cholesterol absorption were also related to fish oil intake. However, to the best of our knowledge, so far there are no indications that fish oils affect cholesterol synthesis or absorption.

To conclude, although this needs further study, we here suggest that it is very likely that in these children prescribed PN and intravenous lipids, changes in serum plant sterol concentrations might have direct effects on endogenous cholesterol synthesis, which may subsequently affect intestinal cholesterol absorption.

## Figures and Tables

**Table 1:** Composition of the soy-based lipid emulsion and the fish-based lipid emulsion.

	SO	FO
Soy bean oil	100%	-
Fish oil	-	100%
Cholesterol (µg/mL)	274.1 ± 3.6	265.0 ± 3.8
Sitosterol (µg/mL)	302.6 ± 2.0	ND
Campesterol (µg/mL)	55.4 ± 30.5	1.0 ± 0.1
Stigmasterol (µg/mL)	65.1 ± 0.5	1.4 ± 0.4

FO: Fish oil (Omegaven); SO: Soybean oil (Intralipid).

**Table:** **Panel** **A**

Sex	Age (mo)	Diagnosis	PN Baseline kkd	EN Baseline kkd	PN 6 mo kkd	EN 6 mo kkd	SO * g/kg/day	FO g/kg/day
Male	1.1	Gastroschisis	91	0	103	0	1	1
Male	3.9	NEC	69	0	61	0	0.9	1
Female	6.8	Gastroschisis	53	37	44	42	0.9	1
Male	2.6	Atresia	89	10	41	61	1	1
Male	2	Long segment Hirschsprung’s	103	7	83	0	2	0.9
Female	42.5	Microvillus Inclusion Disorder	84	0	87	0	0.6	1
Male	76.2	Gastroschisis	66	0	69	0	1.8	1
Male	98.7	MMIHS	66	0	66	0	1.6	1
Male	70.7	Malrotation	58	3.1	58	3	1.3	1

**Table:** **Panel** **B**

Sex	Gestational Age Weeks	PN Baseline	EN Baseline	PN 3 Weeks	EN 3 Weeks	SO Baseline g/kg/day	SO 3 Weeks g/kg/day
Female	31	72	0	106	0	1	2.9
Female	29	56	0	0	86	1	0
Female	29	55	4	0	25	1	0
Female	26	67	0	106	27	1.5	2.9
Male	37	99	0	76	37	1.6	3

SO: soybean oil; FO: fish oil; SBS: short bowel syndrome; PN: parenteral nutrition; EN: enteral nutrition; mo: month; NEC: necrotizing enterocolitis; MMIHS: megacystis microcolon intestinal hypoperistalsis syndrome; kkd: kcal/kg/day. * dose of SO prior to initiation of FO.

**Table 3:** Liver function tests in children treated with fish oil.

	Baseline (on SO)	3 Months (on FO)	6 Months (on FO)
Total bilirubin (mg/dL)	5.5 ± 4.8	2.7 ± 1.3	1.0 ± 0.8 *
Conjugated bilirubin (mg/dL)	4.5 ± 3.8	2.1 ± 0.8 ^#^	0.7 ± 0.5 *
Alanine aminotransferase (IU/L)	128 ± 104	70 ± 53 ^#^	59 ± 55
Aspartate aminotransferase (IU/L)	161 ± 142	80 ± 64 *	67 ± 63 ^#^

* *p* < 0.05 vs. baseline, ^#^ *p* < 0.10 vs. baseline.

**Table:** **Panel** **A**

	Baseline	3 Months	6 Months
	(on SO)	(on FO)	(on FO)
Cholesterol (µmol/L)	3.1 ± 1.2	3.4 ± 1.5	3.0 ± 0.7
Sitosterol (µmol/L)	318.6 ± 176.8	73.3 ± 124.3 *	27.1 ± 42.4 *^$^
Campesterol (µmol/L)	96.7 ± 53.7	31.3 ± 40.7 *	14.6 ± 17.2 *^$^
Stigmasterol (µmol/L)	55.3 ± 36.2	11.0 ± 20.3 *	3.5 ± 5.3 *^$^
Lathosterol (µmol/L)	4.8 ± 3.0	9.9 ± 4.8 *	12.6 ± 8.4 *
Cholestanol (µmol/L)	22.2 ± 6.4	15.7 ± 9.2	11.4 ± 4.6 *

* *p* < 0.05 vs. baseline, ^$^ *p* < 0.05 vs. 3 months.

**Table:** **Panel** **B**

	Baseline (on SO)	3 Months (on FO)	6 Months (on FO)
Sitosterol (µmol/mmol cholesterol)	99.6 ± 24.1	18.3 ± 25.9 *	8.8 ± 13.7 *^$^
Campesterol (µmol/mmol cholesterol)	30.0 ± 7.4	8.1 ± 8.3 *	4.7 ± 5.5 *^$^
Stigmasterol (µmol/mmol cholesterol)	17.0 ± 6.7	2.8 ± 4.3 *	1.2 ± 1.7 *^$^
Lathosterol (µmol/mmol cholesterol)	1.6 ± 0.9	3.0 ± 1.1 *	4.0 ± 2.2 *
Cholestanol (µmol/mmol cholesterol)	7.5 ± 1.3	4.6 ± 2.0 *	3.9 ± 1.6 *^$^

* *p* < 0.001 vs. baseline, ^$^ *p* < 0.05 vs. 3 months.

**Table:** **Panel** **A**

	Baseline	3 Weeks
Cholesterol (mmol/L)	3.11 ± 0.64	2.72 ± 0.29
Sitosterol (µmol/L)	20.5 ± 13.9	91.8 ± 51.0 *
Campesterol (µmol/L)	6.1 ± 4.6	30.5 ± 19.6 *
Stigmasterol (µmol/L)	4.1 ± 2.9	15.1 ± 8.4 *
Lathosterol (µmol/L)	3.0 ± 1.3	4.6 ± 1.1
Cholestanol (µmol/L)	15.9 ± 3.9	9.9 ± 2.8 *

* *p* < 0.05 vs. baseline.

**Table:** **Panel** **B**

	Baseline	3 Weeks
Sitosterol (µmol/mmol cholesterol)	7.0 ± 5.1	32.6 ± 14.6 *
Campesterol (µmol/mmol cholesterol)	2.1 ± 1.7	10.8 ± 5.7 *
Stigmasterol (µmol/mmol cholesterol)	1.4 ± 1.2	5.4 ± 2.5 *
Lathosterol (µmol/mmol cholesterol)	1.0 ± 0.6	1.7 ± 0.5
Cholestanol (µmol/mmol cholesterol)	5.1 ± 0.3	3.6 ± 0.7 *

* *p* < 0.05 vs. baseline.
